# Diabetic ketoacidosis inducing myocardial infarction secondary to treatment with dapagliflozin: a case report

**DOI:** 10.1002/ccr3.858

**Published:** 2017-04-13

**Authors:** José M. Gil‐Perdomo, Tomás F. Fariña González, Benjamín Jordán‐Arias, Sara Domingo‐Marín, Juan J. González Armengol, Fernando Martínez‐Sagasti

**Affiliations:** ^1^ Critical Care DepartmentHospital Clínico San CarlosMadridSpain; ^2^ Emergency DepartmentHospital Clínico San CarlosMadridSpain

**Keywords:** Dapaglifozin, diabetic ketoacidosis, myocardial infarction, pharmacovigilance, sodium‐glucose cotransporter 2 inhibitors, side effects

## Abstract

Sodium‐glucose cotransporter 2 (SGLT2) inhibitors are able to provoke diabetic ketoacidosis (DKA) with absence or low levels of ketone bodies in urine and slightly elevated blood glucose levels, which could delay the diagnosis; however, the presence of high urine output, due to the excretion of glucose, can help to identify the true cause.

## Introduction

Sodium‐glucose cotransporter 2 inhibitors (SGLT2i) are one of the most recently discovered classes of oral antidiabetics. They act by inhibiting the reabsorption of glucose in the luminal membrane of the proximal tubule. Evidence suggests that SGLT2i promote body weight loss, produce a mild reduction in both systolic and diastolic blood pressure, and could decrease disease progression by improving *β*‐cell insulin secretion and insulin sensitivity in peripheral tissues, associated with a reduction in plasma glucose concentration. Although the most frequent side effects of these drugs are minor, such as genital mycotic infections and lower urinary tract infections, several cases of diabetic ketoacidosis (DKA) have been reported in both type 1 diabetes mellitus (T1DM) and type 2 diabetes mellitus (T2DM) patients who were taking SGLT2i, with or without other predisposing factors. We present the case of a male patient who had been switched to a SGLT2i several weeks prior to admission to our hospital because of poor control with metformin monotherapy, who developed DKA associated with an acute coronary syndrome and without other potentially precipitating causes.

## Case Report

A 58‐year‐old male with dyslipidemia, an eight‐year history of T2DM, a family history, his mother, of T2DM, with no known micro‐ or macrovascular complications, was admitted to the emergency department for malaise, epigastric pain, polyuria, and progressive dyspnea which had begun 10 h ago. He had experienced a 2‐kg weight loss over the last few days. His usual medications included aspirin 100 mg q24 h, atorvastatin 40 mg q24 h, and metformin 850 mg q8 h, which had been switched to dapagliflozin 20 days before, due to poor glycemic control, with HbA1c 12% (108 mmol/mol). His vital signs included a heart rate of 122 bpm, respiratory rate 33 rpm, blood pressure 142/70 mmHg, temperature 36.1°C, and body mass index 22.5 kg/m^2^. On physical examination, somnolence, dry skin and mucous membranes, a Kussmaul breathing pattern, and a capillary refill of 3 sec were observed. Blood tests revealed hemoglobin 17.1 g/dL (13.5–18), leukocytes 19.5 × 10^3^ (4–10 × 10^3^), platelets 296 × 10^3^ (150–450 × 10^3^), glucose 248 mg/dL (60–100), creatinine 0.97 mg/dL (0.67–1.17), sodium 136 mmol/L (135–145), potassium 4.7 mmol/L (3.5–5.5), chloride 101 mmol/L (95–112), phosphate 4.9 mg/dL (2.5–4.5), amylase 70 U/L (10–115), lipase 28 U/L (1–67), pH 6.95 (7.35–7.45), pCO_2_ 23 mmHg (35–45), HCO_3_ 5 mmol/L (22–26), lactate 1.8 mmol/L (0–1.5), urine ketone bodies >150 mg/dL (0–0), CK 112 U/L (1–190), CK‐MB 7.3 ng/mL (0.1–5), and troponin I 0.07 ng/mL (0.001–0.05). The electrocardiogram (EKG) showed sinus rhythm with right bundle branch block, and nonspecific repolarization abnormalities. Because of the right bundle branch block was not previously known, a new troponin test was performed six hours later with a peak value of 4.28 ng/mL. Treatment with crystalloids, continuous infusion of intravenous insulin, and administration of potassium and sodium bicarbonate were begun in the emergency room (ER). Due to a poor response over the first two hours, with the persistence of lactic acidosis, the patient was transferred to the intensive care unit (ICU), where more aggressive rehydration with crystalloids was started, without further modifications of the original therapeutic plan.

Two days later, the patient was discharged from the ICU to the endocrinology ward. Because of his coronary risk factors and the elevated troponin on admission, a coronary angiography was performed, showing triple‐vessel disease. Successful bypass surgery without extracorporeal circulation was performed a few days later, with internal mammary artery grafts to the anterior descendent and marginal obtuse arteries and a saphenous vein graft to the right coronary artery. He was discharged 3 days later on Lantus™ Sanofi‐aventis S.p.a Valcanello,03012 Anagni (FR), Italia (insulin glargine) 20 IU and Insulina Novorapid™: Novo Nordisk A/S. Hallas Allé, DK‐4400. Kalundborg, Dinamarca (insulin aspart) 6‐4‐4‐0 IU subcutaneous insulin, aspirin, clopidogrel, enalapril, bisoprolol, atorvastatin, and furosemide. The oral antidiabetic treatment with dapagliflozin was not restarted. During follow‐up by endocrinology, C‐peptide, anti‐GAD, and IA‐2 antibodies were required due to normal BMI and lack of family history suggested T1DM or a latent autoimmune diabetes of adults (LADA) rather than T2DM as etiology of his diabetes. C‐peptide was 1 mg/dL (0.9–7.1) with glycemia 214 mg/dL (60–100), anti‐GAD <5 U/mL (0–12.5), and anti‐IA‐2 <7.5 U/mL (0–7.5).

## Discussion

Inhibition of the reabsorption of glucose in the proximal tubule by SGLT2i leads to urinary excretion of 50–60% of filtered glucose 1. This mechanism of action is glucose‐dependent, becoming negligible when blood glucose drops below 90 mg/dL. Therefore, the risk of hypoglycemia with these oral antidiabetic agents is lower compared to insulin‐dependent antidiabetic drugs 2.

Diabetic ketoacidosis develops in diabetic patients, who suffer an increase in blood ketone bodies as a consequence of both increased production in the liver and a reduced urinary clearance of ketones. This severe acute complication is associated with a 30% rise in mortality 3, and SGLT2 therapy seems to predispose to this, a rare adverse event occurring in up to 1 of 1000 patients 4. Dapagliflozin and empagliflozin have been reported to increase glucagon levels, promoting hepatic ketogenesis. Additionally, some researchers have reported that SGLT2 is expressed in pancreatic alpha cells, contributing in part to the alpha cells’ glucose sensor mechanism, thereby increasing the secretion of glucagon. Studies in animals have demonstrated that glucagon promotes hepatic secretion of kisspeptin‐1, which in turn suppresses glucose‐stimulated endogenous insulin secretion, further increasing the risk for DKA. This has not been shown to occur in humans 5. Nevertheless, the second mechanism involved in the increase in blood ketone bodies is a rise in tubular reabsorption, which could be secondary to inhibition of SGLT2‐mediated Na^+^ reabsorption, increasing the tubular Na^+^ concentration and thus the electronegative gradient, in turn leading to reabsorption of negatively charged ketone bodies.

Ketoacidosis produced by SGLT2i has some particularities, as it can be associated with normal or slightly increased blood glucose levels due to high kidney glucose excretion. Furthermore, increased renal reabsorption may be the mechanism underlying low urine concentrations of ketone bodies, which should preferably be measured in plasma 5. This atypical presentation may delay diagnosis and treatment 6, as in the case of our patient, whose slightly elevated blood glucose levels made emergency doctors prioritize ruling out other causes of lactic acidosis, such as sepsis. This possibility was finally dismissed as the patient presented polyuria and normal blood pressure.

Diabetic ketoacidosis is typically seen in T1DM patients, who have a total deficit of insulin production, but in T2DM patients, it may occur under stressful situations such as trauma, surgery, or infection 5. This patient attended the ER with nonspecific symptoms and an elevated of serum troponin concentration suggestive of a non‐ST‐elevation myocardial infarction. Thus, although Dapagliflozin intake has been reported to cause DKA in a setting of well‐recognized precipitating factors 5, in this patient, the absence of chest pain, with no clear electrocardiographic signs of ischemia and only a minor elevation of serum troponin, does not support that an acute coronary syndrome caused such a severe metabolic disturbance. In fact, tachycardia and tachypnea disappeared a few hours after DKA was corrected, with normalization of troponin levels and without administration of any antianginal drugs. For these reasons, we think that the plausible sequence of events is that DKA acted as a “cardiac stress test,” triggering the acute coronary syndrome as a consequence of an increased oxygen demand. We recognize, however, that the alternative explanation of an acute coronary syndrome causing DKA in a patient already at risk due to Dapagliflozin therapy cannot be completely ruled out. On the other hand, normal BMI at admission and lack of family history suggested T1DM o LADA as possible causes of DM. Nevertheless, antibodies were negative, which rules out autoimmune cause, and basal C‐peptide was low at the presence of hyperglycemia which could act as a confounding factor. Otherwise, patient had lost approximately 20 kg since the diagnosis of DM 8 years ago, and therefore, these findings make us think that long‐term DM2 with insulinopenia is the most likely diagnosis. The European Medicines Agency (EMA) and the FDA have published recommendations for a safer use of SGLT2i, which include suspecting this complication in the presence of symptoms in patients who are taking these drugs, even if blood sugar levels are normal or slightly elevated 4, 6. In this situation, the drug should be stopped and not restarted until another cause has been identified or ketoacidosis has resolved. Healthcare professionals should be cautious when using SGLT2i in patients with risk factors for ketoacidosis and consider stopping therapy before major surgery or if a serious acute illness develops. Although the EMA considers that the benefits of SGLT2i continue to outweigh the risks in the treatment in T2DM 4, we would like to alert about SGLT2 inhibitors being able to cause DKA with undetectable or low levels of urine ketone bodies and only slightly elevated blood glucose, making diagnosis problematic. A high urine output, due to the excretion of glucose, may be a clue to detect undiagnosed DKA.

## Authorship

JMGP: wrote the manuscript. TFFG: reviewed the manuscript and wrote the cover letter. BJA**,** JJGA, SDM, and FMS: reviewed the manuscript.

## Conflict of Interest

None declared.
